# Integrative Analysis of DiseaseLand Omics Database for Disease Signatures and Treatments: A Bipolar Case Study

**DOI:** 10.3389/fgene.2019.00396

**Published:** 2019-04-30

**Authors:** Chun Wu, Bevan E. Huang, Guang Chen, Timothy W. Lovenberg, David J. Pocalyko, Xiang Yao

**Affiliations:** ^1^Computational Sciences, Discovery Sciences, Janssen Research & Development, LLC, Spring House, PA, United States; ^2^Neuroscience Therapeutic Area, Janssen Research & Development, LLC, La Jolla, CA, United States; ^3^Computational Sciences, Discovery Sciences, Janssen Research & Development, LLC, La Jolla, CA, United States

**Keywords:** meta-analysis, transcriptomics, bipolar, mania, depression, omics, human brain

## Abstract

Transcriptomics technologies such as next-generation sequencing and microarray platforms provide exciting opportunities for improving diagnosis and treatment of complex diseases. Transcriptomics studies often share similar hypotheses, but are carried out on different platforms, in different conditions, and with different analysis approaches. These factors, in addition to small sample sizes, can result in a lack of reproducibility. A clear understanding and unified picture of many complex diseases are still elusive, highlighting an urgent need to effectively integrate multiple transcriptomic studies for disease signatures. We have integrated more than 3,000 high-quality transcriptomic datasets in oncology, immunology, neuroscience, cardiovascular and metabolic disease, and from both public and internal sources (DiseaseLand database). We established a systematic data integration and meta-analysis approach, which can be applied in multiple disease areas to create a unified picture of the disease signature and prioritize drug targets, pathways, and compounds. In this bipolar case study, we provided an illustrative example using our approach to combine a total of 30 genome-wide gene expression studies using postmortem human brain samples. First, the studies were integrated by extracting raw FASTQ or CEL files, then undergoing the same procedures for preprocessing, normalization, and statistical inference. Second, both *p*-value and effect size based meta-analysis algorithms were used to identify a total of 204 differentially expressed (DE) genes (FDR < 0.05) genes in the prefrontal cortex. Among these were *BDNF*, *VGF*, *WFS1*, *DUSP6*, *CRHBP*, *MAOA*, and *RELN*, which have previously been implicated in bipolar disorder. Finally, pathway enrichment analysis revealed a role for GPCR, MAPK, immune, and Reelin pathways. Compound profiling analysis revealed MAPK and other inhibitors may modulate the DE genes. The ability to robustly combine and synthesize the information from multiple studies enables a more powerful understanding of this complex disease.

## Introduction

Transcriptomics technologies such as next-generation sequencing (NGS) based RNA-Sequencing (RNA-Seq) and DNA chip based gene expression microarray provide a high-throughput and cost-effective solution to evaluate whole-genome gene expression signatures (Ramasamy et al., 2008; Wu et al., 2017a). These platforms enable researchers to measure tens of thousands of genes simultaneously and have become one of the most widely used approaches in biological research. Detecting differentially expressed (DE) genes or predictive biomarkers is the most common goal in transcriptomics studies. In addition, using large-scale perturbation databases such as Connectivity Map (CMAP) or Library of Integrated Network-based Cellular Signatures (LINCS) (Duan et al., 2016), transcriptomics studies can facilitate the development of pharmacotherapies that modulate disease-associated gene expression signatures (Subramanian et al., 2017). Numerous omics studies on human diseases and animal models are published each year. Despite their great promise, individual studies sharing similar hypotheses may generate results that are not reproducible due to a small number of samples, potential confounding factors, disease heterogeneity, and differences in platforms or bioinformatics methods (Walsh et al., 2015). There is a clear need to effectively manage, integrate, and synthesize the information from related transcriptomics studies to improve our understanding and generate a unified picture of complex diseases.

There are two conceptual frameworks for the integration of information from multiple gene expression studies: cross-platform normalization (merging) and meta-analysis (Taminau et al., 2014). Merging refers to concatenating multiple datasets prior to statistical analysis, while meta-analysis is considered a late stage integration combining statistical results from each individual study (Hamid et al., 2009). The vast quantity of and diverse platforms represented in both NGS- and microarray-based datasets present many challenges for compatibility of data and removal of batch effects, and make meta-analysis a more appealing approach at large-scale (Ramasamy et al., 2008). In the present study, we developed a meta-analysis workflow and applied it to the bipolar disorder. Chang et al. (2013) systematically compared the performance of current meta-analysis methods, including six *p*-value combination methods (Fisher, Stouffer, adaptively weighted Fisher, minimum *p*-value, maximum *p*-value, and rth ordered *p*-value), two combined effect size methods (fixed effects model and random effects model) and four combined ranks methods (RankProd, RankSum, product of ranks and sum of ranks). These methods were categorized into three hypothesis settings (candidate DE genes in “all” [HS*_A_*], “one or more” [HS_B_], or “most” [HS*_r_*] studies) based on their strengths for detecting DE genes (Tseng et al., 2012).

We applied our method to extract insights from existing studies of human postmortem brain tissues in bipolar disorder, which are very heterogeneous and often individually underpowered. Also referred to as manic-depressive disorder, bipolar disorder is a serious mental illness that causes changes in mood, energy, and activity levels (Grande et al., 2016). Based on data from National Comorbidity Survey Replication (NCS-R), the bipolar disorder affects 2.8% of United States adult and 2.9% of United States adolescents (Merikangas et al., 2010). Despite multiple transcriptomics studies of bipolar disorder, a clear understanding of the genomic basis of the disease has not yet emerged. These studies typically use postmortem brain tissues, which is the most relevant for bipolar. However, due to the relative instability of RNA, these gene expression studies are often influenced by factors such as postmortem interval (PMI) (Li et al., 2003), freezer interval and cause of death (Tomita et al., 2004; Wu et al., 2017a). As a result, the genes and pathways identified from individual studies have largely been inconsistent. To address the above issues, we developed a systematic meta-analysis framework and applied it to the largest gene expression datasets in brain tissues from bipolar patients to date.

## Materials and Methods

### Integrate Transcriptomic Studies in the DiseaseLand Database

We adopted Omicsoft methods and used its service to integrate transcriptomics data into the DiseaseLand database^1^. Briefly, we first selected microarray and RNA-Seq platform-based studies in four therapeutic areas: Oncology, Immunology, Cardiovascular and Metabolism, and Neuroscience. Raw data were extracted from public sources such as the Gene Expression Omnibus (GEO^2^) and ArrayExpress^3^, as well as from Janssen internal, collaborators and consortia. These studies were further filtered based on sample size, disease relevance, case and control composition, gene coverage and other factors. Common sample ontologies were applied to name and categorize samples, diseases and treatments. Common gene ontologies were also applied to all platforms to name the same genes in a species and the same ortholog genes across species. Consistent preprocessing, QC, normalization and statistical inference procedures were applied to all studies on the same platforms. This application of common ontologies and consistent data processing enabled searching all studies in the database and retrieving comparable results among studies.

### Identify Suitable Transcriptomics Datasets in Bipolar Disorder

A detailed review protocol was established, which made minor changes to the meta-analysis guidelines suggested in Ramasamy et al. (2008). We searched our database for studies conducted in postmortem human brain tissues using RNA-Seq or genome-wide microarray-based technologies. The resulting study titles, abstracts, and full texts were manually reviewed for potential duplicates. We performed a further literature search on public repositories including ArrayExpress, GEO, Sequence Read Archive (SRA), and Stanley Medical Research Institute to identify any other studies that were potentially missed by the DiseaseLand database, and further identified other unpublished internal data sources for inclusion. In total, 30 RNA-Seq or microarray-based datasets on bipolar disorder were included (Table 1).

**TABLE 1:** Human brain transcriptomic datasets of bipolar.

Study name	Size	Access	Platform name	Region
Internal.caudate	177	Internal	Illumina.HiSeq2500	Striatum
Internal.DLPFC	89	Internal	Illumina.HiSeq2500	PFC
GSE12649.BA46	67	GSE12649	Affymetrix.HG-U133A	PFC
GSE35974.cerebellum	87	GSE35974	Affymetrix.HuGene-1_0-st-v1	Cerebellum
GSE35977.PCX	96	GSE35977	Affymetrix.HuGene-1_0-st-v1	PCX
GSE53239.15433.DLPFC	12	GSE53239	Illumina.HiSeq1000	PFC
GSE53239.9115.DLPFC	10	GSE53239	Illumina.GenomeAnalyzerII	PFC
GSE5388.BA9	61	GSE5388	Affymetrix.HG-U133A	PFC
GSE5389.BA11	21	GSE5389	Affymetrix.HG-U133A	OFC
GSE53987.BA46	36	GSE53987	Affymetrix.HG-U133_Plus_2	PFC
GSE53987.hippocampus	36	GSE53987	Affymetrix.HG-U133_Plus_2	Hippocampus
GSE53987.neostriatum	35	GSE53987	Affymetrix.HG-U133_Plus_2	Striatum
GSE78936.BA11	28	GSE78936	Illumina.HiSeq2000	PFC
GSE78936.BA24	13	GSE78936	Illumina.HiSeq2000	PFC
GSE78936.BA9	13	GSE78936	Illumina.HiSeq2000	PFC
GSE80336.DStriatum	36	GSE80336	Illumina.HiSeq2000	Striatum
GSE80655.ACC	48	GSE80655	Illumina.HiSeq2000	ACC
GSE80655.DLPFC	47	GSE80655	Illumina.HiSeq2000	PFC
GSE80655.NAc	46	GSE80655	Illumina.HiSeq2000	Striatum
GSE81396.caudate	8	GSE81396	Illumina.HiSeq2000	Striatum
GSE81396.putamen	8	GSE81396	Illumina.HiSeq2000	Striatum
NC.hippocampus	29	Neuropathology Consortium	Illumina.HiSeq	Hippocampus
NC.OFC	29	Neuropathology Consortium	Illumina.HiSeq	OFC
NC.PFC	30	Neuropathology Consortium	Illumina.HiSeq	PFC
SAS1.BA46	65	StanleyArrayStudy1	Affymetrix.HG-U133A	PFC
SAS16.thalamus	23	StanleyArrayStudy16	Affymetrix.HG-U133_Plus_2	Thalamus
SAS17.hippocampus	41	StanleyArrayStudy17	Affymetrix.HG-U133_Plus_2	Hippocampus
SAS2.BA46.10	40	StanleyArrayStudy2	Affymetrix.HG-U133A	PFC
SAS4.BA6	27	StanleyArrayStudy4	Affymetrix.HG-U133_Plus_2	PFC
SAS5.BA46	55	StanleyArrayStudy5	Affymetrix.HG-U133_Plus_2	PFC

*Several studies examined tissues from multiple human brain regions.*

### Raw Data Acquisition and Preprocessing

To remove bias introduced by different bioinformatics pipelines used in the original studies, we integrated the raw data by applying a consistent systematic approach to each individual study. For studies carried out with Affymetrix Gene Chip, raw CEL files were extracted from the DiseaseLand database. We applied the RMA method (Irizarry et al., 2003) in Omicsoft, which not only extracts expression data from Affymetrix microarrays, but also carries out background correction, normalization, and summarization. Customized CDF files were used to directly get gene level expression for improving the interpretation and accuracy of the data (Dai et al., 2005). For datasets generated on RNA-Seq platforms, raw fastq files were extracted from DiseaseLand. Bam files were generated using the Omicsoft OSA aligner with human Genome B37 as the reference genome (Hu et al., 2012). We then used the RSEM algorithm (Li and Dewey, 2011) to derive read count values for each gene in UCSC gene model. Genes that have more than 1 cpm (counts per million) in at least 50% of samples were kept. Limma/voom transformation (Law et al., 2014) as applied to generate normalized gene expression matrices. To remove surrogate variables for unknown sources of variation, we performed Surrogate Variable Analysis (SVA) (Leek and Storey, 2007). The top surrogate variables were identified using “leek” method (Leek and Storey, 2007), which were then regressed out to obtain residuals with sources of confounding factors removed.

### Quality Control Process at the Sample- and Study-Level

We applied systematic quality control (QC) processes to all datasets. At the sample level, inter-array correlation (IAC) based QC was performed for each study to identify outlier samples (Oldham et al., 2008). IAC was defined as the Pearson correlation coefficient of the expression levels for a given pair of samples, which provides an unbiased approach to remove samples with divergent gene expression levels. We removed outlier samples that fell below the −3 standard deviation (SD) cutoff. At the study level, any RNA-Seq datasets with median sample alignment rates less than 40% were excluded from the meta-analysis. Also, an unbiased systematic study-level QC was applied to assess the quality of the studies for meta-analysis and determine the final inclusion/exclusion criteria (Kang et al., 2012). Briefly, six quantitative QC measures were evaluated, including IQC (evaluating homogeneity of coexpression structure across studies), EQC (consistency of coexpression information with pathway database), AQCg (accuracy of DE gene detection), AQCp (accuracy of enriched pathway detection), CQCg (consistency of DE gene ranking), and CQCp (consistency of enriched pathway ranking). A standard mean rank (SMR) summary score was calculated based on these six QC measures to identify problematic studies. We then investigated the metadata manually to determine causes for low ranks, including the source of the data, sample size, platform, or other experimental conditions.

### Individual Statistics and Meta-Analysis Approaches

We applied both meta-analysis strategies combining *p*-values and effect sizes across studies. We first calculated *p*-values from non-parametric permutation analysis of a penalized t-statistic in each individual study. We randomly permuted the labels of observations 1,000 times to get adjusted *p*-values. We then applied meta-analysis algorithms, including Fisher, Stouffer, maximum *p*-value (maxP) and r-th ordered *p*-value (rOP) to combine individual *p*-values across studies and generate meta-analyzed *p*-values (Song and Tseng, 2014). The product of ranks (PR) and the sum of ranks (SR) algorithms were implemented to apply a naïve product or sum of the DE evidence ranks across studies. *P*-value combination usually combine two-sided *p*-values, thus a one-sided test correction was also performed to guarantee identification of DE genes with concordant DE direction (Song and Tseng, 2014). To combine effect sizes across studies, the procedure described by Choi et al. (2003) was applied. A random permutation of 1,000 times was implemented to estimate individual effect sizes and FDR. We then fitted a fixed-effect model (FEM) and random-effect model (REM) to combine effect sizes across studies (Choi et al., 2003). The methods used for detecting DE genes in most of the studies were the rth order *p*-value (rOP) and the REM. Tseng et al. (2012) discussed the pros and cons of these methods in a recent review article. In the present study, although multiple methods were tested, “most” or [HS*_r_*] setting was mainly discussed in the example of bipolar disorder.

### Pathway Enrichment Analysis

To get a functional overview of the significant meta-analyzed genes, we performed over-representation tests on shared significant DE genes by using clusterProfiler in R (Yu et al., 2012) and Ingenuity Pathway Analysis (IPA, QIAGEN). Shared significant meta-analyzed DE genes (FDR < 0.05) between rOP and REM were tested against given pathways from disease ontology (DO), Kyoto Encyclopedia of Genes and Genomes (KEGG) databases and IPA. Over-representation test was used to estimate the *p-*values. To correct for multiple comparisons problem, the *q*-values were calculated and reported for FDR control.

### Candidate Compounds Prioritization

We prioritized candidate compounds via anti-genomic similarity between DE genes and compound profiles within LINCS L1000 (Subramanian et al., 2017). Briefly, the upregulated and downregulated DE genes from the meta-analysis were compared against gene expression signatures of small-molecule perturbations from the LINCS L1000 database using Enrichr (Kuleshov et al., 2016). The mechanism of action, target and disease indication for significant compounds (adjusted *p*-value <0.01) were obtained from DrugBank (Wishart et al., 2006) and PubChem. Drugs with unclear pharmacological actions were removed.

### Statistical Analysis and Dataset Access

Raw data from RNA-Seq and microarray were pre-processed by using Omicsoft Array Studio. The statistical computing on analysis-ready gene expression datasets was performed in the R language^4^ (v3.4). The public RNA-Seq and microarray raw datasets can be downloaded from GEO, SRA, or Stanley Medical Research Institute.

## Results

### Integration of Transcriptomic Studies in the DiseaseLand Database

**FIGURE 1:**
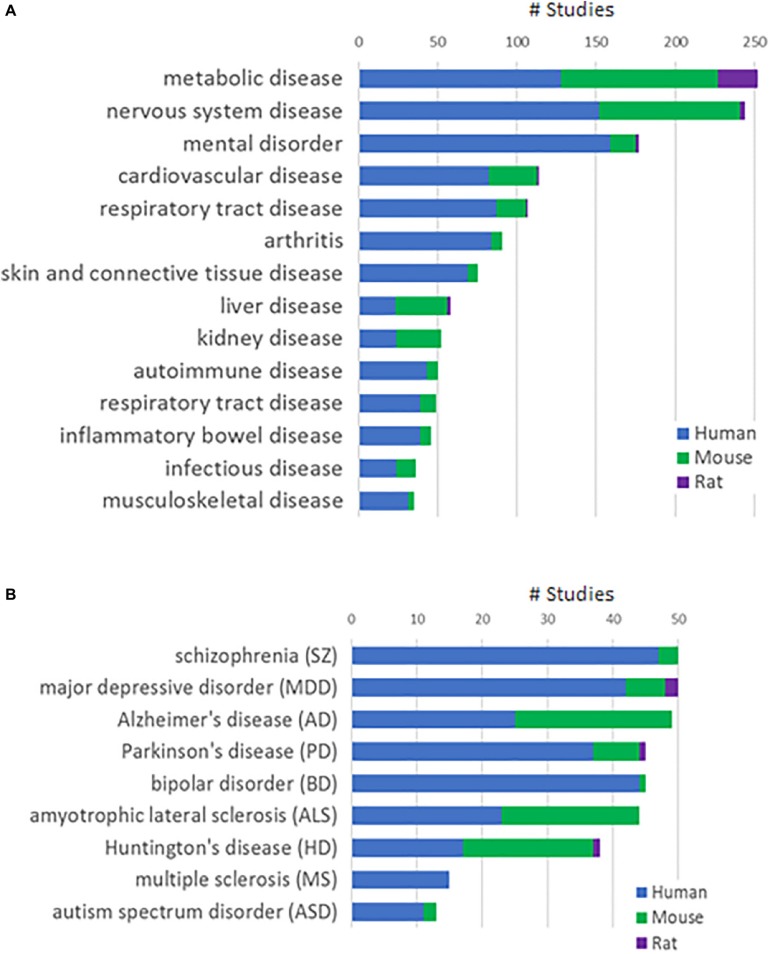
Study collections in the Janssen DiseaseLand database. **(A)** Number of studies in major disease areas. **(B)** Number of studies in major diseases of the mental disorder and nervous system disease areas. Segments in the stacked bars represent parts of studies in human (blue), mouse (green), or rat (purple).

We integrated a total of 1,885 human and 1,460 animal (mouse or rat) studies from public and Janssen internal sources into the Janssen DiseaseLand database. Six hundred and thirty studies are RNA-Seq based and the remaining are derived using microarray-based technology. Figure 1A shows the distribution of studies in various disease areas, including mental disorders, cardiovascular and metabolic diseases and immune diseases. In our collection of main nervous system diseases such as Alzheimer’s disease, Huntington’s disease, and amyotrophic lateral sclerosis, around 50% were conducted in animal models (Figure 1B). In studies of mental disorders like schizophrenia, major depressive disorder, and bipolar disorder, however, there are relatively fewer studies from animal models. This difference may reflect a greater availability of human samples especially post-mortem human brain tissues. It may also reflect that modeling psychiatric disorders in animals is extremely challenging due to the subjective nature of behavior-based diagnostics, the lack of biomarkers and the still developing understanding of relevant neurobiology and genetics (Nestler and Hyman, 2010). Hence in this study, we only included human datasets generated from RNA-Seq and Affymetrix microarray-based platforms in bipolar disorder.

### Workflow for Transcriptomics Data Processing and Meta-Analysis Using DiseaseLand

**FIGURE 2:**
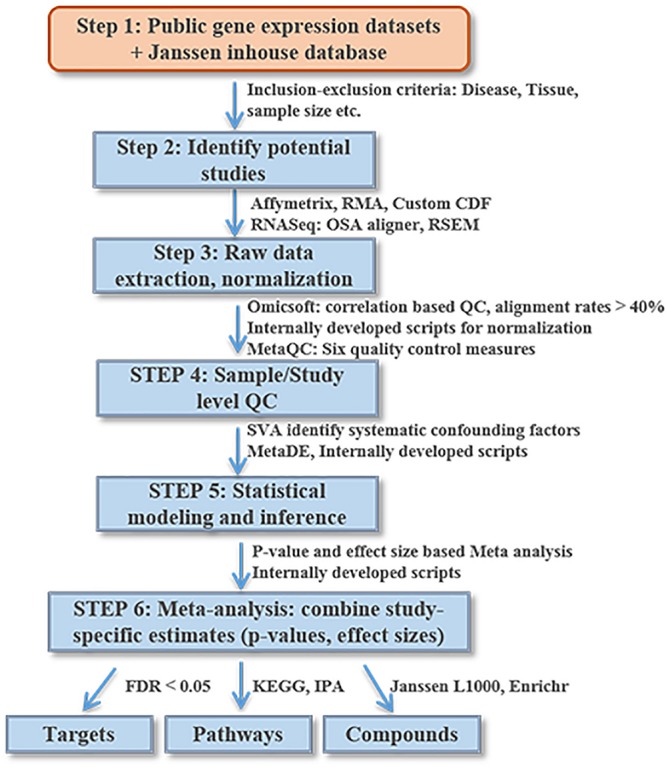
An illustrative diagram of the workflow for meta-analysis of DiseaseLand database. Detailed processes were discussed in the “Materials and Methods” and “Results” sections.

**FIGURE 3:**
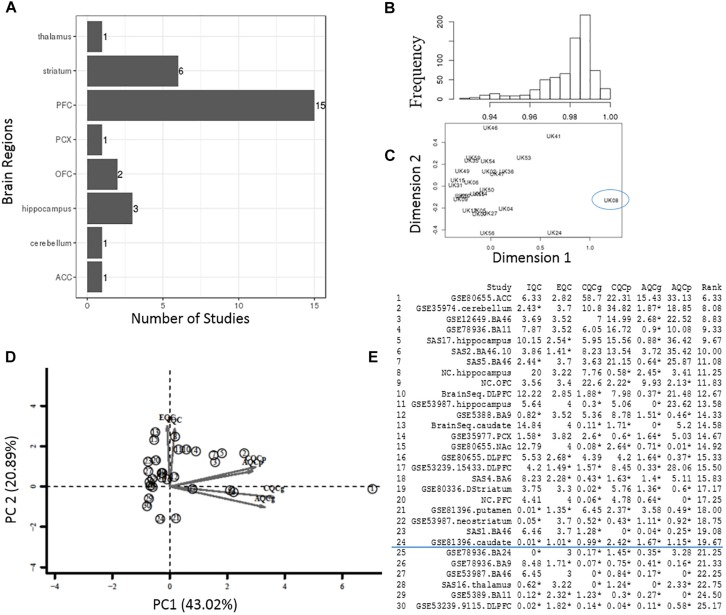
Quality control process at the sample- and study-level. **(A)** The total number of datasets in different brain regions. **(B,C)** Interarray correlations and MDS plots were used to identify potential outlying samples. The frequency distribution plot shows an overall mean IACs of 0.979 in the example StanlyArray4 study. The sample UK08 was flagged as an outlier in both IAC analysis and MDS plot. **(D)** PCA biplot of QC measures in 30 bipolar datasets. The datasets located in the opposite direction of arrows were candidates for problematic studies. **(E)** A total of 30 datasets were ranked by standardized mean rank (SMR) summary score.

Figure 2 lists the analysis framework for integrating transcriptomics datasets from the Janssen DiseaseLand database. In this case study of bipolar, we identified a total of 30 datasets after applying the inclusion–exclusion criteria (Table 1). Raw data were extracted and pre-processed with Omicsoft Array Studio-based pipelines (Methods). Of these 30 datasets, all samples (*n* = 1313) were from post-mortem human brain tissues including the thalamus, striatum, prefrontal cortex (PFC), parietal cortex (PCX), hippocampus, cerebellum, anterior cingulate cortex (ACC) (Table 1 and Figure 3A).

In the sample-level QC step, we calculated the IAC for each individual study to flag potential outlying samples (Methods) (Oldham et al., 2008). As an example, the frequency diagram in Figure 3B shows the distribution of IACs within the Stanley Array Study 4 (SAS4). The overall mean IAC across 27 samples in the SAS4 dataset was 0.979. We removed any samples with mean IACs falling below 3 standard deviations of overall mean IACs, including the sample “UK08” in the example SAS4 dataset (Figure 3C).

In the study-level QC step, we applied an unbiased systematic approach (Kang et al., 2012). Six QC measures and standardized mean rank score, which evaluate the co-expression structure, accuracy/consistency of DE genes or enriched pathways across 30 bipolar datasets, were obtained as described in the “Materials and Methods” section and summarized in Figures 3D,E. The principal components (PC) biplot (Figure 3D) was used to assist the decision for inclusion or exclusion of datasets in the present bipolar meta-analysis. Each study was projected from 6D QC measures to a 2D PC subspace. The datasets located in the opposite direction of arrows were candidates for problematic studies (Kang et al., 2012). Figure 3E lists the detailed QC measures and ranks based on SMR score, a quantitative summary score derived by calculating the ranks of each QC measure. In the present study, 20% of these studies with relative low-ranking scores were removed from meta-analysis.

Individual study analyses were performed to obtain *p*-values and effect sizes, which were used for multiple meta-analysis approaches. Pathway enrichment analyses were then conducted on the genes identified as significantly DE through meta-analysis. A disease-associated DE gene signature was also used for prioritization of candidate compounds via comparation between disease signature and compound profiles within the LINCS L1000 (Kuleshov et al., 2016). The presented framework is general and can be applied to datasets from any complex diseases.

### Comparison of Meta-Analysis Approaches in Bipolar Disorder

**FIGURE 4:**
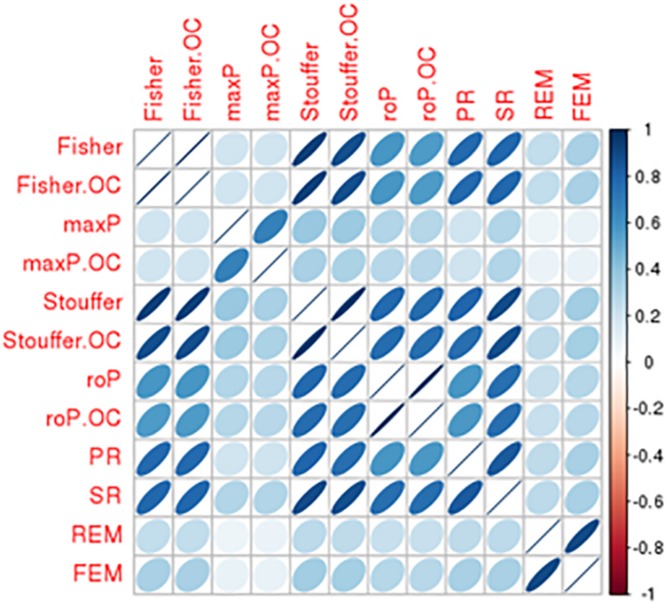
Comparison of DE results generated from multiple meta-analysis approaches in bipolar. Correlation matrix shows the Spearman’s correlations among *p*-values from Fisher/Fisher.OC, maxP/maxP.OC, Stouffer/Stouffer.OC, roP/roP.OC, PR, SR, REM, and FEM approach.

There was a total of 9,310 common genes in each individual dataset after pre-processing. Table 2 shows the number of significant DE genes with FDR < 0.05 generated by using multiple meta-analysis methods. Under algorithms that detecting DE genes with non-zero effect sizes in one or more studies (HS_B_), we got a total of 3,133 to 6,552 significant DE genes; while under the HS_A_ hypothesis which detects DE genes with non-zero effect sizes in all studies, only 11 to 58 genes fall below the FDR cut-off. For the downstream analysis, we decided to choose significant meta-analyzed DE genes (FDR < 0.05) under HS*_r_* hypothesis (rOP and REM), which identifies DE genes with non-zero effect sizes in most studies. Although the number of DE genes with FDR < 0.05 varies, the *p*-values generated by these multiple approaches are highly correlated (Figure 4), suggesting concordant results are generated by these multiple algorithms.

**TABLE 2:** Number of significant meta-analyzed DE genes by using multiple approaches.

Approaches	Targeted HS	Combining statistics	FDR < 0.05
PR	*HS_A_*	Rank	15
SR	*HS_A_*	Rank	11
maxP	*HS_A_*	*P*-values	58
maxP.OC	*HS_A_*	*P*-values	24
rOP	*HS_r_*	*P*-values	1366
rOP.OC	*HS_r_*	*P*-values	960
REM	*HS_r_*	Effect sizes	1166
Fisher	*HS_B_*	*P*-values	6552
Fisher.OC	*HS_B_*	*P*-values	6151
Stouffer	*HS_B_*	*P*-values	4514
Stouffer.OC	*HS_B_*	*P*-values	4402
FEM	*HS_B_*	Effect sizes	3133

**HS*_A_, DE genes with non-zero effect sizes in all studies; *HS_B_*, DE genes with non-zero effect sizes in one or more studies; *HS_r_*, DE gene with non-zero effect in “majority” of studies.*

Based on the available datasets in specific brain regions, we carried out separate meta-analyses for studies conducted in the PFC (*N* = 15) or striatum (*N* = 6). Common significant DE genes (FDR < 0.05) under both algorithms of HS*_r_* hypothesis (rOP, REM) were reported. Supplementary Tables S1–S3 lists 327 DE genes in any regions and 204 in the PFC and 49 in the striatum regions. We decided to focus on studies of the PFC because this is arguably the most relevant region for bipolar.

### Pathway Enrichment Analysis and Compounds Prioritization for Bipolar

**FIGURE 5:**
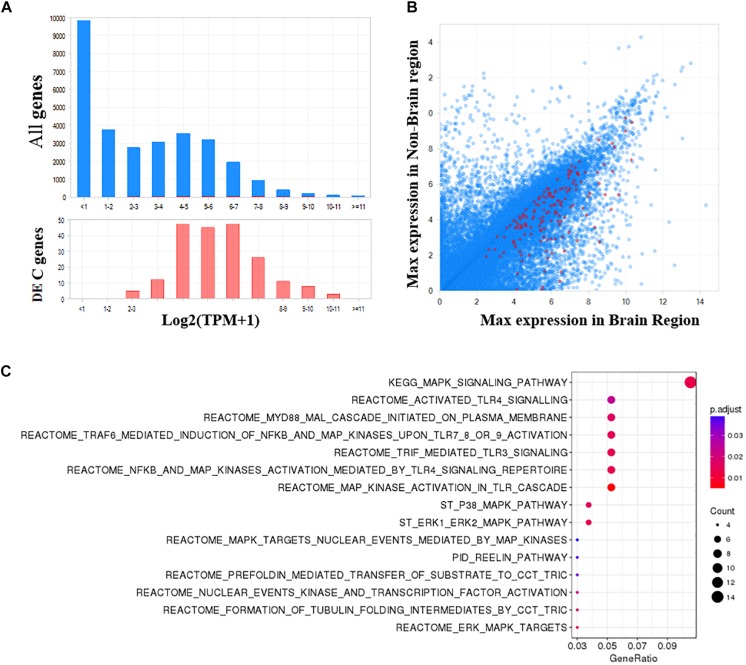
Functional analysis of significant DE genes in the PFC of bipolar. **(A)** The frequency distribution plots of gene expression in brain shows that the significant DE genes identified in PFC are more abundant in brain, and **(B)** more specific to brain compared to all other genes. **(C)** Pathway enrichment analysis using MSigDB (Canonical pathways) shows a total of 15 pathways are significantly enriched in bipolar (FDR *q*-value <0.05).

As shown in Figure 5A, the 204 DE genes have a higher expression in brain regions compared with all human genes. Additionally, these genes are generally more expressed in the brain than non-brain regions (Figure 5B). To obtain a functional overview of these significant meta-analyzed DE genes in the PFC of individuals with bipolar, we conducted overrepresentation tests on pathway databases including the MSigDB, gene ontology (GO) and DO. As shown in Figure 5C and Supplementary Table S4, these genes were significantly enriched in a total of 15 pathways from MSigDB (FDR < 0.05), including MAPK signaling related pathways and the reelin signaling pathway. Using the GO database (biological process), we identified 33 significantly enriched categories (Supplementary Table S5). Among them, brain development, MAPK signaling, and angiogenesis processes were dysregulated in bipolar. Although not significant after multiple test correction, these DE genes showed an enrichment in mental depression (DOID:1596, *p*-value = 0.004), mood disorder (DOID:3324, *p*-value = 0.005), and schizoaffective disorder (DOID:5418, *p*-value = 0.01) (Supplementary Table S6).

Compounds significantly associated with an increase or decrease in bipolar-associated gene expression changes were listed in Supplementary Tables S7, S8 (adjusted *P*-value <0.01). Table 3 summarizes the top compounds that appear more than five times in multiple cell lines and compound doses. Interestingly, many of them are targets of the MAPK signaling related pathway, the most significant hit in the pathway enrichment analysis. These results not only confirmed previous findings but also revealed novel biological mechanisms in bipolar disorder.

**TABLE 3:** List of significant compounds that could modify the expression of bipolar signature genes.

Compounds	SigDown0L1000Up	SigUp0L1000Down	Sum	Mechanism
CGP-60474	19	1	20	cdk1/cdk2 inhibitor
Dasatinib	8	11	19	Bcr-Abl tyrosine-kinase inhibitor
WH-4-025	4	11	15	Lck and Src inhibitor; also inhibits SIK
KIN001-043	2	13	15	GSK3/WNT inhibitor
Mitoxantrone	10	1	11	Topoisomerase II inhibitor
Crizotinib	6	3	9	c-MET/ALK inhibitor
Radicicol	7	2	9	Hsp90 inhibitor
PI-103	8	1	9	PI3K, mTOR and DNA-PK inhibitor
GSK-1059615	6	3	9	PI3K inhibitor
MK-2206	8	1	9	AKT inhibitor
JW-7-24	7	2	9	LCK inhibitor
Geldanamycin	5	3	8	Hsp90 inhibitor
JNK-9L	8	0	8	JNK inhibitor
A443654	8	0	8	AKT inhibitor
AT-7519	7	0	7	CDK inhibitor
BMS-387032	7	0	7	CDK inhibitor
Alvocidib	7	0	7	CDK9 inhibitor
Vorinostat	6	1	7	HDAC inhibitor
GSK-2126458	6	1	7	mTOR/PI3K inhibitor
AZD-7762	5	1	6	CHK inhibitor
NVP-TAE684	2	4	6	ALK inhibitor
AKT-inhibitor	5	1	6	AKT inhibitor
Torin-2	2	4	6	mTOR inhibitor
Saracatinib	6	0	6	Bcr-Abl tyrosine-kinase inhibitor
Canertinib	2	4	6	pan-erbB tyrosine kinase inhibitor

### Consistent Results With Independent Datasets in Bipolar Disorder

**FIGURE 6:**
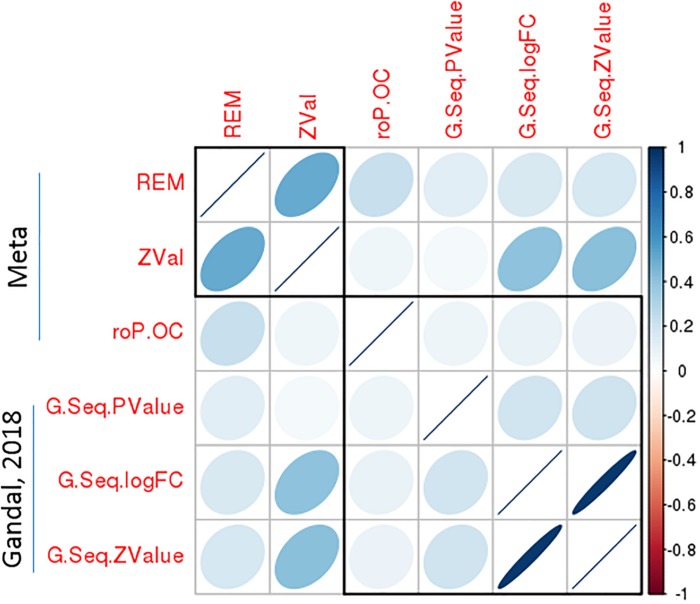
A significant correlation of bipolar-associated gene expression changes between the present meta-analysis study and an independent dataset. Correlation matrix shows the Spearman’s correlations between each pair of *p*-values from the current meta-analysis (REM and roP.OC) and Gandal’s study, as well as correlations between *Z*-values of the meta-analysis and logFC/*Z*-values of Gandal’s study. Among these, a high Spearman’s correlations (rho = 0.41) was observed between *Z*-values from the meta-analysis and logFC/*Z*-values from Gandal’s study.

To validate the significant bipolar-related gene expression changes, we compared our findings with Seifuddin’s mega-analysis study in bipolar, published in 2013 (Seifuddin et al., 2013), and a recently published study (Gandal et al., 2018). The first study considers 10 microarray-based studies with data on 211 bipolar and 229 control samples. However, only 11 genes survived correction for multiple testing with *q*-value <0.05 in the PFC. Supplementary Table S9 shows that nine of these genes were also significantly dysregulated in our analyses by using either the rOP.oc or REM models. Importantly, in all cases, the direction of gene expression changes was identical. In the most recently published study by Gandal et al. (2018), a meta-analysis of CommonMind and BrainGVEX RNA-Seq datasets for bipolar was performed. As shown in Figure 6, the DGE summary statistics from Gandal’s study are consistent with our meta-analysis results. In particular, the logFC or *Z*-value from Gandal’s study was highly correlated with our *Z*-value with Spearman’s rho equals 0.41. These results suggested a consistency of meta-analyzed bipolar-related gene expression changes among the three integrative studies.

## Discussion

We presented here an integrative analysis framework of the Janssen DiseaseLand transcriptomics database, which currently includes 1,885 human and 1,460 animal (mouse or rat) studies from public and Janssen internal sources, and is constantly growing. By applying standardized re-processing to raw datasets, removing outlying samples and problematic datasets, and comparing multiple meta-analysis approaches, we were able to generate a unified gene expression signature for a disease.

We demonstrated our approach on a meta-analysis of 30 gene expression datasets from human brain tissues in bipolar. Bipolar is a serious mental illness with considerable public health implications. However, our understanding of biological mechanisms of bipolar remain frustratingly limited in part due to difficulty in accessing human brain samples (Wu et al., 2017a). Many individual bipolar transcriptomics studies contain only tens of samples (Table 1), which may contribute to a lack of reproducibility in genes and pathways identified from each study. By combining these smaller studies through meta-analysis, we can additionally reduce the effects of heterogeneity in platforms and pipelines among studies. By applying our proposed meta-analysis framework to a total of 30 existing studies of bipolar in the human brain, we generated a highly reliable gene signature that is associated with bipolar.

While our studies encompassed tissue from several brain regions, we saw little pathway enrichment in regions outside of the PFC due to the smaller number of studies from these tissues. The PFC plays crucial roles in cognitive functions and has been implicated in many psychiatry disorders, and indeed we found 204 significant DE genes (Supplementary Table S2). Interestingly, these genes were enriched not only in bipolar, but also in mood disorders generally (Supplementary Table S5). Of these (Supplementary Table S2), *PDE4B* (*Z*-value = −3.04), *RELN* (*Z*-value = −4.59), *WFS1* (*Z*-value = 2.83), *DUSP6* (*Z*-value = −3.63), *MAOA* (*Z*-value = 5.14), *CRH* (*Z*-value = −4.46), *FKBP5* (*Z*-value = 5.55), *EDN1* (*Z*-value = 5.64), *CRHBP* (*Z*-value = −2.99) were significantly DE in bipolar. The phosphodiesterase 4B (*PDE4B*) was found to be involved in cognitive function in animal models and serves as a susceptibility gene for bipolar disorder and schizophrenia (Kahler et al., 2010). Reelin (*RELN*) plays a significant role in the development of the brain, and connections have been seen between RELN dysfunction and psychiatric disorders (Ovadia and Shifman, 2011). Wolfram syndrome gene (*WFS1*) has been indicated to play a role in the susceptibility for mood disorders. Koido et al. (2005) suggested a possible relation between polymorphisms in *WFS1* and increased risk for mood disorders. Dual specificity phosphatase 6 gene (*DUSP6*), also known as mitogen-activated protein kinase phosphatase 3 (MKP3), plays a key role in regulating members of mitogen-activated protein (MAPK) kinase superfamily. Lee et al. (2006) showed a genetic association of *DUSP6* with bipolar and its effect on ERK activity. Monoamine oxidase A (MAOA) catalyzes the oxidative degradation of amines, such as dopamine, norepinephrine, and serotonin (Wu et al., 2017b). An association between MAOA polymorphic markers and bipolar disorder was also reported (Rubinsztein et al., 1996). Upregulation of MAOA in the present study suggested an impaired neurotransmission in bipolar. Corticotropin-releasing hormone (*CRH*) and corticotropin-releasing hormone binding protein (*CRHBP*) are peptides involved in the stress response and hypothalamic-pituitary axis regulation. Abnormalities in CRH secretion have been documented in bipolar disorder (Stratakis et al., 1997). FK506-binding protein 51 (*FKBP5*), an important modulator of stress responses, is another gene having genetic association and other evidence for bipolar and other psychiatric diseases (Willour et al., 2009; O’Leary et al., 2013). All these results confirmed previous results regarding bipolar disorder, demonstrating the robustness of our meta-analysis workflow.

Several significantly implicated signaling pathways (Supplementary Table S4) are related to responses to inflammation and immune insults. IL17RB (encoding interleukin 17 receptor B) ranked number two in expression increase in disease (Supplementary Table S2). Expression of IRAK1 (encoding interleukin 1 receptor associated kinase 1) is significantly downregulated (Supplementary Table S2). These data support the role of immune dysfunction as a contributor of bipolar disorder pathology and targeting immune dysregulation for developing bipolar treatment as suggested in the literature (Goldsmith et al., 2016; Mechawar and Savitz, 2016; Rosenblat, 2019).

The meta-analysis also provided evidence for dysfunction of neurotrophic signaling pathways in bipolar disorder (Supplementary Table S4). The data (Supplementary Table S2) also showed expression dysregulation of FGFR3 (encoding fibroblast growth factor receptor 3) and FGF2 (fibroblast growth factor 2). These data support the dysregulation of neurotrophic MAPK signaling in mood disorders (Kyosseva et al., 1999; Einat et al., 2003a, 2003b; Lee et al., 2006). Neurotrophic signaling MAPK pathways are involved in the regulation of neurodevelopmental abnormalities of the brain in psychiatric diseases. It has been suggested that lithium and valproate (VPA), at therapeutically relevant concentrations, robustly activate the ERK MAPK cascade in cultured cells and in the PFC and hippocampus (Hao et al., 2004; Chen and Manji, 2006; Schloesser et al., 2008). Ketamine produces rapid, robust and sustained antidepressant action in patients with treatment-resistant-depression (TRD) and bipolar TRD (Zarate et al., 2006; Diazgranados et al., 2010). Preclinical study showed single ketamine activated neurotrophic signaling including ERK, and promoted synaptic growth, genesis and function (Li et al., 2010, 2011). Given the results that the neurotrophic MAPK pathway is among the most significant finding from our pathway analyses, this may represent a novel target for the development of improved therapeutics for bipolar disorder.

Also implicated in the pathway analysis was the reelin pathway. Among these, both *RELN* (*Z*-value = −4.59) and its receptor *ApoER2* (*LRP8, Z*-value = −2.89) were downregulated in the PFC of bipolar patients. It was known that reelin was involved in neuronal migration, cell aggregation, and dendrite formation. Genetics studies have also reported that the *RELN* is associated with multiple neurological diseases including bipolar disorder, schizophrenia, autism spectrum disorder, and Alzheimer’s disease (Ovadia and Shifman, 2011; Wang et al., 2014; Bufill et al., 2015; Li et al., 2015). Moreover, *ApoER2* was confirmed as a risk gene for psychosis (Li et al., 2016). These findings suggest that reelin and molecules in its downstream signaling pathway could be potentially useful as targets of therapeutical intervention for bipolar disorder.

Another group of pathways implicated in current meta-analysis is those involved in cellular structure formation (Supplementary Table S4). The data (Supplementary Table S5) also showed further supports for the involvement of neuronal developmental genes in bipolar disorders. It is noteworthy that lithium promotes hippocampal neurogenesis (Chen et al., 2000). Neurons derived from induced pluripotent stem cells (iPSC) originated from bipolar patients showed molecular and cellular changes and the changes are differentially revered by lithium in neurons from lithium responding and non-responding bipolar patients (Mertens et al., 2015; Tobe et al., 2017; Stern et al., 2018). The role of neuronal development in bipolar disorder is an emerging field to be further investigated.

A growing body of studies supports the use of large-scale perturbation databases, such as the LINCS for developing novel therapeutic intervention strategies (Hurle et al., 2013). A high-reliability disease gene expression signature is essential for implementing the systems biology approaches. Using the unified bipolar disorder signature and LINCS or Janssen L1000 database, we identified a list of small molecules that could modulate bipolar gene signature (Table 3 and Supplementary Tables S8, S9), which could be further evaluated for disorder modeling and intervention evaluation, and ultimately drug development. It is noteworthy that whether the modulation of bipolar disease genes in the LINCS cellular perturbation assay can be translated to human, including compounds passing the blood–brain barrier, should be evaluated before using the experiments.

While our analyses revealed a unified gene expression signature for the bipolar disorder, there are several limitations to this study. The primary limitation to the generalization of these results is a lack of replication. In the case study of bipolar disorder, individual studies do not have large sample sizes due to lack of accessibility to healthy and diseased human brain tissues. Limited overlaps of DE genes were observed across individual studies, and the same would be expected if trying to replicate meta-analysis results in individual studies. An alternative approach of dividing the datasets into discovery and replication meta-analysis might decrease the power of the analysis. Furthermore, although we applied a systematic study-level QC to exclude low quality datasets, we acknowledge the heterogeneity of the studies and the potential bias associated with the different platforms or brain regions. Despite these limitations, to our knowledge this is the largest gene expression meta-analysis of bipolar disorder using postmortem human brain tissues. As more disease datasets are published and integrated into our database, an incremental refinement by adding new datasets into the meta-analysis will further improve our results (Brown and Peirson, 2018).

In summary, we have collected comprehensive transcriptomics datasets across neuroscience, immunology, oncology and cardiovascular and metabolism disease areas. We established systematic meta-analysis approaches, which can be applied in multiple disease areas to create a unified picture of the disease signature and prioritize drug targets, pathways, and compounds. To illustrate the proposed workflow, we have generated a highly reliable gene signature in bipolar disorder, which confirmed previous theories in bipolar and revealed novel targets that could be potentially useful as new therapeutic treatment strategies.

## Author Contributions

CW, BH, and XY conceptualized the study. CW and XY performed the computational analysis, and prepared the tables and figures. All authors contributed to the interpretation of analytic results and writing the manuscript.

## Conflict of Interest Statement

The authors declare that the research was conducted in the absence of any commercial or financial relationships that could be construed as a potential conflict of interest.

## Footnotes

1 https://www.omicsoft.com/diseaseland/

2 https://www.ncbi.nlm.nih.gov/geo/

3 https://www.ebi.ac.uk/arrayexpress/

4 https://www.r-project.org/
